# Increased antimicrobial resistance in bacterial pneumonia among Egyptian Children during the COVID-19 pandemic

**DOI:** 10.1186/s43168-023-00193-7

**Published:** 2023-03-20

**Authors:** Mahitab Morsy Hussein, Malak Ali Shaheen, Abdelrahman Mahmoud Sleem, Fatma Mostafa Mahmoud, Sally Raafat Ishak

**Affiliations:** 1 Pediatrics Department, Faculty of Medicine, Ain Shams University, Cairo, Egypt; 2 Faculty of Medicine, Alexandria University, Cairo, Egypt; 3 Medical Microbiology and Immunology, Faculty of Medicine, Ain Shams University, Cairo, Egypt

**Keywords:** Pneumonia, COVID-19, Antimicrobial resistance, Egypt

## Abstract

**Background:**

Pneumonia is the major cause of morbidity and mortality among children worldwide. During the COVID-19 pandemic, the use of antibiotics increased which led to the development of antibiotic-resistant strains of pathogenic organisms causing pneumonia in children. So, studies should be directed to register antimicrobial resistance in each country and to develop local antimicrobial stewardship. This study aimed to identify the distribution of bacteria causing pneumonia among Egyptian children in the year 2020 and their antimicrobial sensitivity. A cross-sectional study was done, it included fifty immunocompetent children with pneumonia admitted to Children's Hospital, Ain-Shams University from June 2020 to December 2020. Bacterial cultures were done on sputum collected using cough swab, or endotracheal tube aspirate, with their antimicrobial sensitivity.

**Results:**

Thirty children had Community-acquired pneumonia (CAP), while twenty had Hospital-acquired pneumonia (HAP). Streptococcus pneumonia was the most frequently cultured organism in CAP group 7/30 (23.3%). The sensitivity results found linezolid (50%), followed by fluoroquinolones to be the least resistant. While in the HAP group, Klebsiella pneumonia 9/20 (45%) was the most common organism. Colistin (90%) followed by tigecycline (50%), Amikacin (35%), fluoroquinolones (25%), gentamicin (25%), and imipenem (20%) had the least resistance in the HAP group.

**Conclusion:**

No pathognomonic shift of the bacteria that causes pediatric pneumonia was detected. Although, an increase in antimicrobial resistance was noticed.

## Introduction

The prescription of antibiotics increased during the COVID-19 pandemic to avoid bacterial superinfection that may increase the risk of mortality. Also, the difficulty in differentiation between bacterial and viral infections even by the commonly used biomarkers increased the use of antibiotics during that era [1, 2].

Pneumonia is the leading cause of mortality in children worldwide despite the advances in prevention, diagnosis, and management. Globally, Streptococcus Pneumoniae is the commonest cause of community-acquired pneumonia (CAP), while Gram-negative organisms are the leading cause of Hospital-acquired pneumonia [3].

Multi-drug resistant organism is threatening the whole world nowadays, which is estimated to be 3.3% to 7.6% in CAP in Europe [4]. Also, difficulty in obtaining samples from the infected lungs in children increased the use of empirical antibiotics and delay the diagnosis of the causative organism [3, 5].

Moreover, the decreased effectiveness of conventional antibiotics after the COVID-19 pandemic increased the burden on the scientific community to provide new antibiotics. Studies are now conducted worldwide to register antibiotic resistance to develop local antimicrobial stewardship protocols. This is of utmost importance in developing countries where infection control methods and management resources are limited [6].

This work aimed to investigate antimicrobial susceptibility patterns among bacteria isolated from respiratory tract of children hospitalized with pneumonia amid COVID-19 pandemic.

## Methods

This was a cross-sectional study. It included a consecutive sample of 50 children hospitalized with pneumonia, recruited from the Children’s Hospital, Ain Shams University, Cairo, Egypt, during the period from June 2020 to December 2020. They were subdivided into 30 patients with community-acquired pneumonia and 20 patients with hospital-acquired pneumonia.

Children aged 1 month to 16 years were included. Pneumonia diagnosis and severity grading was applied according to British thoracic society guidelines [7]. Radiological evidence was included to confirm pneumonia diagnosis. It was defined as community-acquired (CAP) if infection was acquired outside hospital or hospital-acquired (HAP) if patient developed clinical and radiological signs of pneumonia 48 h or more after admission to hospital which was not present before [8].

Patients with positive results for a swab for SARS-coV-2 infection, alternative respiratory infections as acute bronchiolitis and tuberculosis, underlying co-morbidities as immunodeficiency, chronic illnesses or chest problems, and ventilator-associated pneumonia were excluded from the study.

All patients were subjected to detailed history taking, laying stress on demographics and prior antibiotic administration. General and local examination was done, including vital data. Pulse oximetry was used to monitor oxygen saturation.

Before enrollment, all patients performed a routine nasal and throat swab to exclude SARS-coV-2 (COVID-19) co-infection. Chest x-ray was done, and findings were interpreted according to WHO standardized criteria for pneumonia diagnosis in children [9].

Routine investigations as complete blood count (CBC) with differential count and C-reactive protein (CRP) were collected from patient’s record using a standardized collection form.

Respiratory specimens were collected for all patients under aseptic conditions using appropriate personal protective equipment within the first 24 h following admission for CAP cases and confirming pneumonia diagnosis for HAP cases. Cough swab was performed for 12 patients (24%) with moderate pneumonia admitted to hospital wards [10]. Endotracheal aspiration was performed for 38 patients (76%) with severe pneumonia admitted to pediatric intensive care unit before mechanical ventilation. Samples were collected in sterile containers and transferred immediately to lab, where they were examined under light microscopy after applying a Gram stain. Then, inoculated on blood agar and chocolate agar in an atmosphere of 5% CO2 and on MaConkey agar at 37˚ C for 24–48 h. The pattern of antimicrobial sensitivity was done by VITEK II.

Statistical analysis of the data.

Based on Mansour and Bendary [11] and Elseify et al. [12], sample size of 50 patients was estimated for 80% statistical power and 0.05 level of significance. Data were fed to the computer and analyzed using IBM SPSS software package version 20.0. (Armonk, NY: IBM Corp) Qualitative data were described using number and percent. The Shapiro–Wilk test was used to verify the normality of distribution Quantitative data were described using range (minimum and maximum), mean, standard deviation, median, and interquartile range (IQR). The significance of the obtained results was judged at the 5% level.

The used tests were: Chi-square test (for categorical variables, to compare different groups. Fisher’s Exact (correction for chi-square when more than 20% of the cells have an expected count less than 5). Student t-test (for normally distributed quantitative variables, to compare between two studied groups). Mann–Whitney test (for abnormally distributed quantitative variables, to compare between two studied groups).

## Results

Thirty of them met the CAP definition and twenty were HAP. Among them, 57% were males while 43% of them were females. The range of age was from 2 months old up to 12 years old with a mean of about 4.5 years. The demographic and laboratory data of the studied children are presented in (Table 1, 2).

**Table 1:** Demographic and clinical characterstics of Egyptian children with pneumonia

	**Patients**	
	**CAP** **(*n* = 30)**	**HAP** **(*n* = 20)**
**Sex**		
Male	15 (50.0%)	12 (60.0%)
Female	15 (50.0%)	8 (40.0%)
**Age (years)**		
Min. – Max	1.5 ms – 12.0	2 ms – 12.0
Mean ± SD	4.33 ± 3.57	4.72 ± 4.16
**Prior Antibiotics use**		
No	10 (33.3%)	0 (0.0%)
Yes	20 (66.7%)	20 (100.0%)
**Heart rate (beat/min)**		
Range	90.0 – 180.0	75.0 – 150.0
Mean ± SD	125.30 ± 24.89	111.25 ± 18.84
**Respiratory rate**		
Range	38.0 – 80.0	28.0 – 75.0
Mean ± SD	53.90 ± 11.60	50.70 ± 11.80
**Temperature (**^**o**^**C)**		
Range	36.50 – 40.20	37.80 – 40.0
Mean ± SD	38.65 ± 0.91	38.95 ± 0.56
**O**_**2**_** saturation**		
Range	85.0 – 94.0	85.0 – 98.0
Mean ± SD	90.40 ± 2.51	92.70 ± 3.25

*CAP* Community-acquired pneumonia, *HAP* Hospital-acquired pneumonia

**Table 2:** Laboratory characteristics of Egyptian children with pneumonia

	**Patients**	
	**CAP** **(*n* = 30)**	**HAP** **(*n* = 20)**
**TLC ( 10**^**9**^** cell/L)**		
range	3.0 -33.0	3.70—33.90
Mean ± SD	18.84 ± 5.62	17.92 ± 7.47
**ANC (10**^**9**^**/L)**		
Range	1.9- 26	1.9—30
Mean ± SD	14.5 ± 4.1	13.2 ± 6.3
**ALC (10**^**9**^**/L)**		
Range	0.23–11.6	1.61–14.4
Mean ± SD	3.69 ± 2.204	3.88 ± 2.95
**Hemoglobin (mg/dL)**		
Range	5.2–13	7–13
Mean ± SD	10.4 ± 1.9	10.3 ± 1.6
**Platelets ( 10**^**9**^** cell/L)**		
Range	55–736	23–598
Mean ± SD	281 ± 146.2	147 ± 140
**CRP (mg/dL)**		
Range	48.0 – 286.60	48.0 – 150.0
Mean ± SD	111.39 ± 67.0	80.75 ± 27.92

*CAP* Community-acquired pneumonia, *HAP* Hospital-acquired pneumonia, *TLC* Total leucocytic count, *ANC* Absolute neutrophilic count, *ALC* Absolute lymphocytic count, *CRP* C-reactive protein, n = number of patients, *SD* Standard deviation

The most common bacteria causing CAP was streptococcus pneumoniae, followed by staphylococcus aureus (16.7%), Hemophilus influenza (10%), and E.coli (10%). While the most common bacteria causing HAP was Klebsiella(45%), followed by Acinetobacter species (20%), E.coli (15%), and Pseudomonas (15%) (Table 3).

**Table 3:** Frequency of isolated bacterial pathogen among pneumonia patients

Isolated bacterial pathogen	Type	
	**CAP** **(*n* = 30)**	**HAP** **(*n* = 20)**
Gram positive		
Streptococcus pneumoniae	7 (23.3%)	0 (0.0%)
Staphylococcus aureus	5 (16.7%)	0 (0.0%)
Gram negative		
Pseudomonas aeruginosa	0 (0.0%)	3 (15.0%)
Klebsiella pneumoniae	2 (6.7%)	9 (45.0%)
Haemophilus influenzae	3 (10.0%)	0 (0.0%)
Enterobacter spp.	1 (3.3%)	0 (0.0%)
Escherichia coli	3 (10.0%)	3 (15.0%)
Acinetobacter spp.	1 (3.3%)	4 (20.0%)

*CAP* Community-acquired pneumonia, *HAP* Hospital-acquired pneumonia

The most sensitive antibiotics to organisms causing CAP was Linezolid (50%), Levofloxacin (36.7%), Ciprofloxacin (33.3%), and Vancomycin (33.3%). While those most sensitive in HAP were Colistin (90%), Tigecycline (50%), Amikacin (35%), Levofloxacin, and Ciprofloxacin (25%) (Table 4).

**Table 4:** Antimicrobial sensitivity pattern among the studied pneumonia children

Sensitivity	Type	
	**CAP** **(*n* = 30)**	**HAP** **(*n* = 20)**
**Clindamycin**	4 (13.3%)	1 (.0%)
**Colistin (Colistimethate Sodium)**	5 (16.7%)	18 (90.0%)
**Co-trimoxazole**	9 (30.0%)	1 (5.0%)
**Vancomycin**	10 (33.3%)	1 (5.0%)
**Linezolid**	15 (50.0%)	1 (5.0%)
**Piperacillin-Tazobactam**	4 (13.3%)	2 (0.0%)
**Azithromycin**	6 (20.0%)	0 (0.0%)
**Clarithromycin**	1 (3.3%)	0 (0.0%)
**Ampicillin-Sulbactam**	6 (20.0%)	3 (15.0%)
**Amoxicillin-Clavulanic acid**	4 (13.3%)	0 (0.0%)
**Amikacin**	2 (6.7%)	7 (35.0%)
**Gentamicin**	2 (6.7%)	5 (5.0%)
**Cefepime**	9 (30.0%)	2 (0.0%)
**Cefoprazone**	7 (23.3%)	2 (0.0%)
**Ceftriaxone**	9 (30.0%)	0 (0.0%)
**Cefotaxime**	8 (26.7%)	0 (0.0%)
**Ceftazidime**	6 (0.0%)	1 (5.0%)
**Cefadroxil**	4 (13.3%)	0 (.0%)
**Cefuroxime**	1 (3.3%)	1 (5.0%)
**Ciprofloxacin**	10 (3.3%)	5 (25.0%)
**Levofloxacin**	11 (6.7%)	5 (25.0%)
**Meropenem**	3 (10.0%)	1 (5.0%)
**Imipenem-Cilastatin**	3 (10.0%)	4 (20.0%)
**Tigecycline**	4 (3.3%)	10 (50.0%)
**Teicoplanin**	5 (16.7%)	0 (0.0%)

*CAP* Community-acquired pneumonia, *HAP* Hospital-acquired pneumonia

The sensitivity to Linezolid ranged from 66.6% to 100% in infections by Streptococcus Pneumonaie, Staphylococcus aureus, and Hemophilus influenza. While, the sensitivity to Colistin ranged from 66.6% to 100% in infections by Acinetobacter, Pseudomonas, E.coli, and Hemophilus influenza (Table 5).

**Table 5:** Distribution of antimicrobial senstivity according to the causing bacterial pathogen and pneumonia group

Type	Organism	Antibiotics	%
CAP	S. pneumoniae	Linezolid	100
		Vancomycin	85.7
		Cefepime	71.4
		Cefotaxime,Ceftriaxone	57.1
		Co-trimoxazole	57.1
	S. aureus	Linezolid	100
		Vancomycin, Clindamycin,	80
		Co-trimoxazole, Levofloxacin, Ciprofloxacin	60
	H. influenzae	Colistin (Colistimethate Sodium)	66.6
		Linezolid	66.6
		Piperacillin-Tazobactam	66.6
		Tigecycline	66.6
HAP	Klebsiella pneumoniae	Colistin (Colistimethate Sodium)	88.8
		Tigecycline	44.4
		Amikacin	33.3
	Acinetobacter SPP	Colistin (Colistimethate Sodium)	100
		Levofloxacin	75
		Amikacin, Ciprofloxacin, Tigecycline	50
	Pseudomonas aeruginosa	Colistin (Colistimethate Sodium)	100
		Amikacin, Gentamicin, Ciprofloxacin, Levofloxacin, Imipenem-Cilastatin	33.3
	E. Coli	Imipenem-Cilastatin, Colistin (Colistimethate Sodium), Tigecycline	100%
		Gentamicin	

*CAP* Community-acquired pneumonia, *HAP* Hospital-acquired pneumonia

## Discussion

There is an increase in the rate of infection by Strep. Pneumoniae over the last years in Egypt even before the COVID-19 pandemic as the pneumococcal vaccine is still not obligatory in Egypt according to the study done by Draz et al. during 2008–2011 that found that the annual rate of detection of Streptococcus pneumonia infection was 54.5/100, 000, with a high case fatality rate (33.3%) [13].

A previous study done in the Children’s Hospital, Ain Shams University during 2012–2013 by El-Seify et al. revealed that the most common cause of bacterial pneumonia was Staphylococcus aureus (13.3%) followed by Streptococcus pneumoniae (7.8%) [12]. Also, the study by Hatem et al. in 2019 found that staphylococcus aureus was the commonest organism causing pneumonia in Egyptian infants and preschool children, followed by Klebsiella (8.33%), Pseudomonas (4.16%) in contrast to our study where Streptococcus pneumoniae was the most common (23.3%) followed by staphylococcus aureus (16.7%) [14].

Going with these results, Fu et al. in 2021, amid of COVID-19 pandemic, 288,377 bacterial isolates were collected from 11 tertiary care children’s hospitals in China found S. pneumoniae (22.5%) to be the most common isolated organism from lower respiratory tract samples [15].

Haemophilus influenza (12.91%) was the most isolated organism in the study by Su et al. in 2021 in China but those with severe pneumonia only were included. In this study, Klebsiella pneumoniae was the most common bacterial cause of HAP (9/20, 45%) [16]. That is in concordance with what Wang et al. found in 2010 in China [17]. Also in agreement with our results, Mansour and Bendary in a previous study on HAP found Acinetobacter spp. (28%) and Klebsiella pneumoniae (20%) were the most common bacterial pathogens that were isolated and cultured [11]. Camomot and Bongo 2010 in the Philippines found that Pseudomonas (21%) followed by Klebsiella 13% and E-coli 10% were the most common organisms causing HAP [18]. Also, Sergevnin et al. (2022) found Klebsiella pneumoniae to be the primary infectious agent that causes pneumonia in hospitalized children [19]. On the contrary, Shahid et al. 2020 found coagulase-negative staphylococcus (CNS) followed by Staphylococcus haemolyticus, Micrococcus species, Acinetobacter, Pseudomonas, and Escherichia coli to be the most common bacterial pathogens identified among the children with HAP. So, our study did not detect a pathognomonic shift of the causative organisms of bacterial pneumonia [20].

Although, WHO (World Health Organization) recommends parenteral ampicillin (or penicillin) and gentamicin as first-line treatment for children aged 2–59 months with severe pneumonia [21]. Moreover, NICE (National Institute for Health and Care Excellence) guidelines in CAP suggest Amoxicillin-clavulanate as the first line in severe CAP [22]. In the current study, Amoxicillin-clavulanate was found to be sensitive in 13% of culture results due to either previous use or resistant organism. We also found that the most commonly used first-choice antibiotics in the case of CAP like cephalosporins or broad-spectrum penicillins were the least sensitive. It also found that linezolid (50%) followed by levofloxacin (36.7%), ciprofloxacin (33.3%), and Vancomycin (33.3%) are the antibiotics that came more frequently in the antibiotic sensitivity of cultures attained from CAP group. In agreement with the current study, Su et al. 2021 found no resistance to Levofloxacin, Vancomycin, and Linezolid in CAP [15]. In this study, Ampicillin–Sulbactam was found to be sensitive only in 20%, while gentamicin was in 6.7% of the isolated organisms in CAP only.

In the current study, HAP was mainly caused by gram-negative organisms that were susceptible to Colistin (90%). In concordance with Ergul et al. study in 2017 that found the isolates were mainly gram-negative, and sensitive to Colistin (92.6%) [23]. Hormozi et al. in 2018 found less resistance to Colistin in the gram-negative isolates [24]. In disagreement with our results, Shahid et al. study in 2021 found the most sensitive antibiotics were levofloxacin and aminoglycosides in HAP culture isolates [20]. However, that study did not include Colistin in antibiotic sensitivity results.

Twenty of the CAP group 20/30 (66.7%) used antibiotics before presenting to the hospital while the entire HAP group 20/20 was on antibiotics due to suspected HAP, sepsis, or treatment of the previous infection. PERCH (Pneumonia Etiology Research for Child Health) project found that antibiotic exposure affects the detection of bacterial pathogens in children with pneumonia [25]. Ericson et al. found that most patients were started on antibiotics for suspected HAP or undifferentiated sepsis in a study in 2020 on hospital-acquired pneumonia in concordance with the present study [8].

Limitations of this study include the small sample size, limited resources, and single-center study that make the results not generalizable. A large multi-center study needs to be done to help generalize the results and make Egyptian guidelines for the treatment of pediatric bacterial pneumonia.

## Conclusion

The distribution of the organisms causing bacterial pneumonia in Egypt during the COVID-19 pandemic did not change from the global distribution as Streptococcus Pneumonaie was the major cause of CAP, and Klebsiella was the major cause of HAP. But, the antimicrobial sensitivity and resistance differ. The least resistant antibiotics in CAP were Linezolid and Fluoroquinolones, while in HAP the least resistant antimicrobials were Colistin and Tigecycline.

## Data Availability

Data are available upon request. The details of the study and procedures were explained to all individuals and their parents, and informed consent was obtained from their legal guardians before enrollment in the study. This study was approved by the Research Ethics Committee, Faculty of Medicine, Ain Shams University (FMASU 731MS/2020/2021).

## Abbreviations

CAP: Community-Acquired Pneumonia
CNS: Coagulase-Negative Staphylococcus
COVID-19: Coronavirus Disease -2019.
HAP: Hospital- Acquired Pneumonia
NICE: National Institute for Health and Care Excellence
PERCH: Pneumonia Etiology Research for Child Health
WHO: World Health Organization
